# Preparation of Peptide-Based Magnetogels for Removing Organic Dyes from Water

**DOI:** 10.3390/gels10050287

**Published:** 2024-04-24

**Authors:** Farid Hajareh Haghighi, Roya Binaymotlagh, Paula Stefana Pintilei, Laura Chronopoulou, Cleofe Palocci

**Affiliations:** 1 Department of Chemistry, Sapienza University of Rome, Piazzale Aldo Moro 5, 00185 Rome, Italy; 2 Research Center for Applied Sciences to the Safeguard of Environment and Cultural Heritage (CIABC), Sapienza University of Rome, Piazzale Aldo Moro 5, 00185 Rome, Italy

**Keywords:** magnetogels, magnetic γ-Fe_2_O_3_ nanoparticles, peptide-based hydrogels, water purification, methyl orange, methylene blue, rhodamine 6G

## Abstract

Water pollution by organic dyes represents a major health and environmental issue. Despite the fact that peptide-based hydrogels are considered to be optimal absorbents for removing such contaminants, hydrogel systems often suffer from a lack of mechanical stability and complex recovery. Recently, we developed an enzymatic approach for the preparation of a new peptide-based magnetogel containing polyacrylic acid-modified γ-Fe_2_O_3_ nanoparticles (γ-Fe_2_O_3_NPs) that showed the promising ability to remove cationic metal ions from aqueous phases. In the present work, we tested the ability of the magnetogel formulation to remove three model organic dyes: methyl orange, methylene blue, and rhodamine 6G. Three different hydrogel-based systems were studied, including: (1) Fmoc-Phe_3_ hydrogel; (2) γ-Fe_2_O_3_NPs dispersed in the peptide-based gel (Fe_2_O_3_NPs@gel); and (3) Fe_2_O_3_NPs@gel with the application of a magnetic field. The removal efficiencies of such adsorbents were evaluated using two different experimental set-ups, by placing the hydrogel sample inside cuvettes or, alternatively, by placing them inside syringes. The obtained peptide magnetogel formulation could represent a valuable and environmentally friendly alternative to currently employed adsorbents.

## 1. Introduction

Safe water is a global need for all aspects of life (e.g., household, industrial, and agricultural purposes) and, considering the current limitation of water resources, the importance of effective water recycling and purification is constantly growing [1]. Commonly used technologies for wastewater treatment are helpful but should be improved in terms of their economic feasibility, efficiency, and environmental footprint [2]. Due to the absence of adequate wastewater treatment methods, different dangerous materials constantly enter natural water resources [3]. Every year, tons of chemically stable synthetic dyes are wasted from the pharmaceutical [4,5], garment [6], textile [7], leather, ink, paper [8], and plastic industries [9], negatively affecting aquatic life and also threatening human health, as many of these chemicals are highly hazardous and have been classified as mutagenic, carcinogenic, or genotoxic [10]. Furthermore, these water contaminants decrease sunlight penetration into bodies of water, which influences photosynthetic processes and affects aquatic flora and fauna [11]. In particular, sunlight plays a crucial role in the photosynthesis of aquatic plants. It provides the energy needed for the conversion of inorganic carbon to organic carbon compounds, which is the fundamental biological process on Earth. Synthetic dyes such as methylene blue (MB), rhodamine 6G (Rh6G) (model cationic dyes), and methyl orange (MO) (a model anionic dye) cause several health disorders like respiratory tract infections, skin disease, and eye irritation, so their removal from water resources is necessary [12].

There are standard methods for wastewater treatment, including chemical precipitation, adsorption, ion exchange, electrochemical separation, and coagulation-flocculation methods. Nonetheless, all of these methods have some drawbacks, e.g., high energy consumption, partial contaminant elimination, high operation costs, and the production of toxic sludges [13]. Today, the removal of contaminants from wastewater is considered a crucial step towards achieving sustainable development. Among the approaches listed [14], adsorption is still the most commonly used method, and an ideal adsorbent removes most pollutants and can be recovered easily using cost-effective methods like electrochemical or solvent treatments [15,16]. However, common commercial adsorbents such as biochar, zeolites, and activated carbon pose a high risk of water contamination by themselves.

Recently, hydrogel nanohybrids have become increasingly used in numerous adsorption-based water treatment applications [17]: generally, a polymer forms a hydrophilic porous network and nanomaterials are used as modifiers to enhance the adsorption properties [18]. For water remediation, the possibility of using biocompatible materials is very important. Peptide hydrogels can be considered as promising candidates to decrease the self-contamination risk of adsorbent systems [19]. Compared with commercially available adsorbents, these hydrogels benefit from being porous and highly hydrophilic, as well as from possessing large surface areas and numerous functional groups. In particular, short self-assembling peptides are an interesting class of biocompatible and biodegradable hydrogel-forming materials. Their synthesis is generally simple and scalable. Moreover, both the properties and self-assembly of peptides can be tuned by altering the peptide sequence [20].

Magnetic nanoparticles (NPs) can be incorporated into hydrogels to provide a magnetic property to the resulting hybrid system. Among magnetic nanostructures, magnetite (Fe_3_O_4_) and maghemite (γ-Fe_2_O_3_) NPs have been extensively used for environmental applications because of their biocompatibility, well-assessed synthesis methods, and on/off superparamagnetic properties [21]. Although γ-Fe_2_O_3_NPs show a slightly smaller magnetic moment than Fe_3_O_4_NPs, they are more stable in air [22], and more reliable for practical applications. To date, various nanohybrid-based γ-Fe_2_O_3_NPs have been used as adsorbents to remove several organic dyes (e.g., MO, rose bengal, MB, brilliant cresyl blue, Congo red, thionine, and Janus green B) from aqueous solutions with significant adsorption efficiencies. Aside from the numerous advantages of peptide-based hydrogel nanohybrids, their poor mechanical properties and low elasticity are their two main limitations in water remediation applications; however, these drawbacks may be overcome by using appropriate cross-linkers.

In a recent work [23], we reported the preparation of a new peptide composite magnetogel made of a Fmoc-Phe_3_ hydrogel matrix containing γ-Fe_2_O_3_-polyacrylic acid (PAA) NPs (γ-Fe_2_O_3_NPs) and its application for removing Cr(III), Ni(II), and Co(II) from water, demonstrating that both the native hydrogel as well as the magnetogel can effectively eliminate all the examined metal ions from water. Moreover, thanks to the presence of γ-Fe_2_O_3_NPs, the efficiency of this system can be promoted by the application of a magnetic field. In the present study, we tested the ability of the magnetogel formulation to remove three model dyes: MO, MB, and Rh6G. On this basis, three different hydrogel-based systems, including (1) Fmoc-Phe_3_ hydrogel (gel); (2) γ-Fe_2_O_3_NPs loaded in the hydrogel (γ-Fe_2_O_3_NPs@gel); and (3) γ-Fe_2_O_3_NPs@gel in the presence of an external magnetic field (γ-Fe_2_O_3_NPs@gel + mf), were studied. The removal efficiency of these gels was evaluated using two experimental set-ups, by placing hydrogel samples: (1) inside cuvettes or (2) inside syringes.

## 2. Results and Discussion

### 2.1. Preparation of γ-Fe_2_O_3_NPs and Magnetogels

The magnetogel nanohybrids were synthesized in two steps, as described in our previous publication [23]. Firstly, PAA-stabilized γ-Fe_2_O_3_NPs were prepared by a co-precipitation method to obtain small and colloidally stable NPs. Co-precipitation is an uncomplicated and cost-effective way to prepare γ-Fe_2_O_3_NPs, and involves starting from aqueous solutions of Fe(II) and Fe(III) and adding a base as a precipitating agent. This method requires mild temperatures and no organic solvents or toxic precursors, and allows for the preparation of large amounts of NPs. Also, thanks to its simpleness, reliability, and environmentally friendly conditions, it can be adjusted and scaled up to an industrial scale.

For the magnetogel synthesis, γ-Fe2O3NPs were suspended in an aqueous phase containing the hydrogel precursors and the gelation process was carried out. The main advantage of such a blending method is its simplicity; however γ-Fe2O3NPs must be stabilized (i.e., with PAA) to prevent their heterogeneous distribution within the hydrogel network. The hydrogel formation was conducted using a biotechnological approach that employs a microbial lipase to catalyze the formation of the Fmoc-Phe_3_ hydrogelator, as previously reported [24]. The resulting magnetogel was characterized in a previous work through FT-IR, Raman, and XPS experiments [23].

### 2.2. Rheology and Swelling Ability Studies

**Figure 1:**
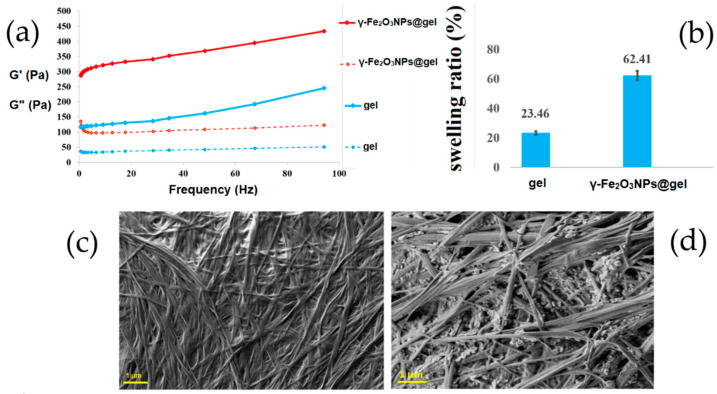
(**a**) Frequency sweep of the gel and γ-Fe_2_O_3_NPs@gel; (**b**) swelling abilities of the gel and γ-Fe_2_O_3_NPs@gel; SEM images of: (**c**) the gel and (**d**) γ-Fe_2_O_3_NPs@gel.

Rheological studies were conducted to unravel the influence of the modifiers used on the tensile strength of peptide hydrogels. The presence of γ-Fe_2_O_3_NPs was able to enhance the rheological properties of the hydrogel, as previously reported (Figure 1a).

After the addition of γ-Fe_2_O_3_NPs to the pristine hydrogel, its swelling ability increased up to ≈62%, which may be related to the presence of NPs that provide porosity inside the hydrogel, increasing its swelling properties (Figure 1b).

### 2.3. SEM Experiments

SEM was employed to investigate the morphology of the pristine gel as well as that of the magnetogel. Figure 1c shows the typical fibrillary structure of the pristine hydrogel. The fibrils are several micrometers long while their width is between 100 and 150 nm. The addition of magnetic NPs to the gel did not seem to have any significant effect on its fibrillary structure (Figure 1d). On the basis of SEM experiments, the average size of γ-Fe_2_O_3_NPs was determined to be in a range between 15 and 60 nm.

### 2.4. Removal Studies of MB, Rh6G and MO from Water

**Figure 2:**
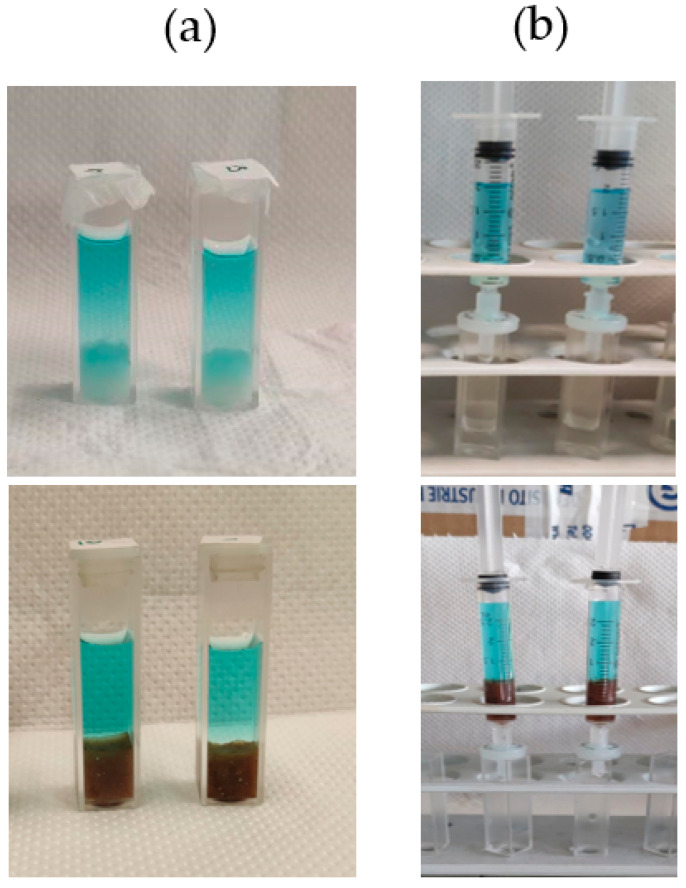
The two different set-ups used for the removal studies of organic dyes from water: (**a**) employing cuvettes and (**b**) employing syringes.

Two different experimental set-ups were developed for the removal of MB, Rh6G, and MO by using different hydrogel samples (Figure 2). In the first set of experiments, the gel samples were prepared inside cuvettes, then the aqueous dye solution was placed on top of them. With this configuration, the dyes diffuse into the gel matrix by gravity (without the need to apply any external forces). In the other set of experiments, the gel samples were prepared inside syringes. In the removal experiments, the dye solutions were allowed to flow inside the syringes, placed vertically, either by gravity or by applying an external force. Therefore, in this set-up, the dyes pass through the gel matrix and exit from the lower inlet of the syringe.

#### 2.4.1. Experimental Set-Up Employing Cuvettes

**Figure 3:**
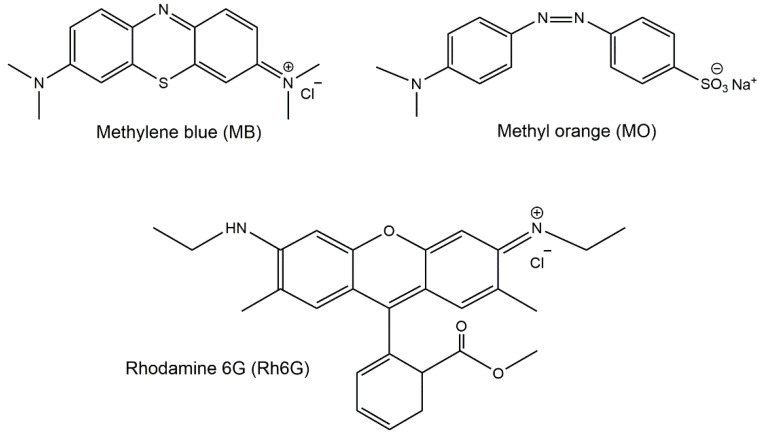
Chemical structures of methylene blue (MB), rhodamine 6G (Rh6G) (model cationic dyes), and methyl orange (MO) (model anionic dye).

MB has a heterocyclic aromatic structure (Figure 3) with characteristic absorbance peaks at 293 and 664 nm, related to *π*—π* and *n*—π* transitions, respectively [25]. The visible absorption (664 nm) was monitored during the MB removal studies. Regarding Rh6G, it contains a xanthene moiety that is responsible for its *π*—π* absorption at visible wavelengths [26]. The visible absorption at 527 nm was monitored to evaluate the Rh6G removal by the adsorbents. The anionic dye MO has a strong absorption at around 500 nm, which was selected for the adsorption experiments.

Three different adsorbents were used to remove the three organic dyes described above from aqueous solutions: the native gel, γ-Fe_2_O_3_NPs@gel, and γ-Fe_2_O_3_NPs@gel + mf (mf: external magnetic field).

**Figure 4:**
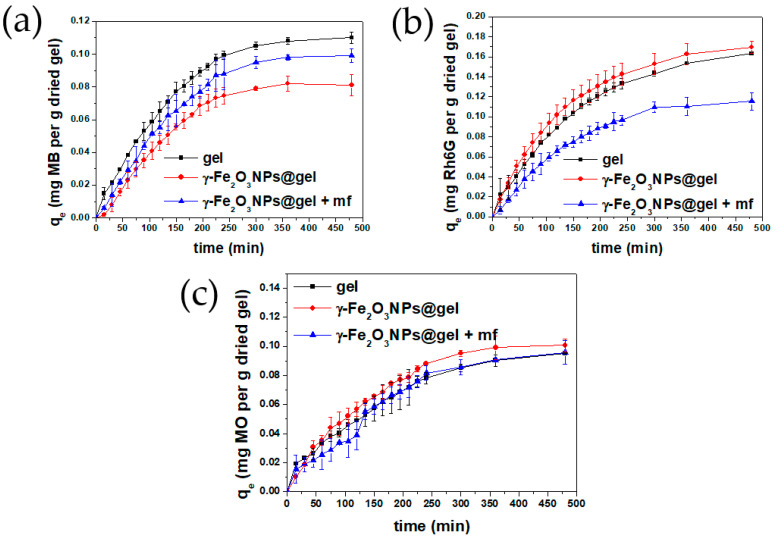
Adsorption capacities (q_t_, mg/g) of the native gel, γ-Fe_2_O_3_NPs@gel, and γ-Fe_2_O_3_NPs@gel + mf, versus time for MB (**a**), Rh6G (**b**), and MO (**c**).

In particular, the adsorption abilities of these materials were evaluated at different contact times with the solution of the selected dye and expressed as q_t_ (mg of dye absorbed by 1 g of dry hydrogel). The evolution of the q_t_ values as a function of time for each dye + adsorbent system is reported in the plots in Figure 4a–c, for contact times ranging from 0 to 480 min. Also, Table 1 and Table 2 show the removal efficiencies/capacities and calculated kinetic parameters of the three adsorbent systems for the three dyes, respectively.

The results obtained with this experimental set-up show that the magnetic NPs change the adsorption properties of the pristine gels and, also, that the addition of an external magnetic field additionally modifies the adsorbing character of the gels. Over the whole removal process for the three examined systems, no significant changes were detected in the maximum wavelengths of the dyes and, as can be seen in Figure 4a–c, the dye adsorption decreased with time. This general feature, observed for all dyes and adsorbents, is probably linked to the progressive saturation of the adsorbing sites of the gels over time, which eventually reaches equilibrium after approximately 400 min.

Figure 4a shows that the q_t_ values for MB follow the trend gel > γ-Fe_2_O_3_NPs@gel + mf > γ-Fe_2_O_3_NPs@gel, which is different from those observed for Rh6G and MO, where γ-Fe_2_O_3_NPs@gel exhibits the best performance in terms of dye removal (Figure 4b,c). Such a difference may be ascribed to the different structures of the dyes, which affect the gel–dye interaction at the molecular level.

Another result worth mentioning is the changes in removal efficiencies (RE%) and final adsorption capacities for different systems, summarized in Table 1. For MB, the results show that the removal efficiencies of γ-Fe_2_O_3_NPs@gel and γ-Fe_2_O_3_NPs@gel + mf are decreased by 26.3% and 10.1%, respectively, in comparison with the native gel. For Rh6G, both magnetogels and pristine gels show similar RE% values, and the combination of magnetogels with the application of a magnetic field significantly drops the adsorption efficiency by 29.1%. For MO, the magnetogel shows the highest RE% value, while the interaction of magnetogels with the magnetic field increases the dye removal by 16.7%.

Regarding the different effect of γ-Fe_2_O_3_NPs on dye adsorption, it is in good agreement with data from the literature on magnetogels [27,28] and is due to the ability of NPs embedded in hydrogels to affect both the cross-linking degree and porosity of the gels, influencing the surface channels and, therefore, the entry, exit, and adsorption of molecules [29]. Also, the application of an external magnetic field can further change the adsorption, because magnetogels are able to exert an on/off effect on the hydrogel pores [30]. The magnetic dipole-dipole orientation of γ-Fe_2_O_3_NPs towards the external magnetic field is able to affect the swelling and shrinking of γ-Fe_2_O_3_NPs@gels [31], most probably affecting the permeability of dyes into the gel network [32,33].

As is well known, the study of adsorption kinetics is able to provide information on the nature of the adsorption (e.g., physisorption or chemisorption) [34]. In this study, we used pseudo-first- and pseudo-second-order kinetic models [22], as described in the Supplementary Materials. The obtained kinetic parameters are summarized in Table 2, where the best correlations (in terms of R^2^) of MB and Rh6G studies were mainly obtained when using the pseudo-first-order kinetic model, although not for γ-Fe_2_O_3_NPs@gel (Rh6G) studies. For this system, the equilibrium adsorption capacity, q_e_, obtained from the pseudo-first-order relation was more similar to the experimental values reported in Figure 4 and Table 1, suggesting that the adsorption of Rh6G onto these adsorbents mainly occurs through physisorption. MB and Rh6G dyes possess cationic imine and amine groups that can favor their adsorption on the hydrogels, thanks to different electrostatic interactions that may occur, such as that between the positively charged imine nitrogen of the dyes and the negatively charged hydroxyl groups of the hydrogel. Moreover, H-bonding interactions among the amine group of the dyes and the hydroxyl groups of the hydrogels may be established [35]. Returning to the possible pseudo-second-order mechanism estimated for γ-Fe_2_O_3_NPs@gel (Rh6G), it is attributed to chemisorption, which may depend on the chelation between the carboxyl and amine groups of the gels (–NH, –OH, and –COOH) and the lone pair electrons of the dye molecules [36,37,38,39,40,41]. Conversely, to these cationic dyes, MO (as an anionic dye) shows much lower RE% and q_e_ values, which may be related to its negative charge significantly limiting its electrostatic interaction with the gels, even though it was used at higher initial concentrations (10 ppm) than MB and Rh6G (5 ppm). For MO, two adsorbents show physisorption (gel and γ-Fe_2_O_3_NPs@gel + mf), controlled by physical forces like dipole–dipole interactions, hydrogen bonds, van der Waals forces, and hydrophobic interactions. Based on these results, the prepared adsorbent is more suitable for the removal of cationic dyes, rather than anionic ones.

#### 2.4.2. Experimental Set-Up Employing Syringes

**Figure 5:**
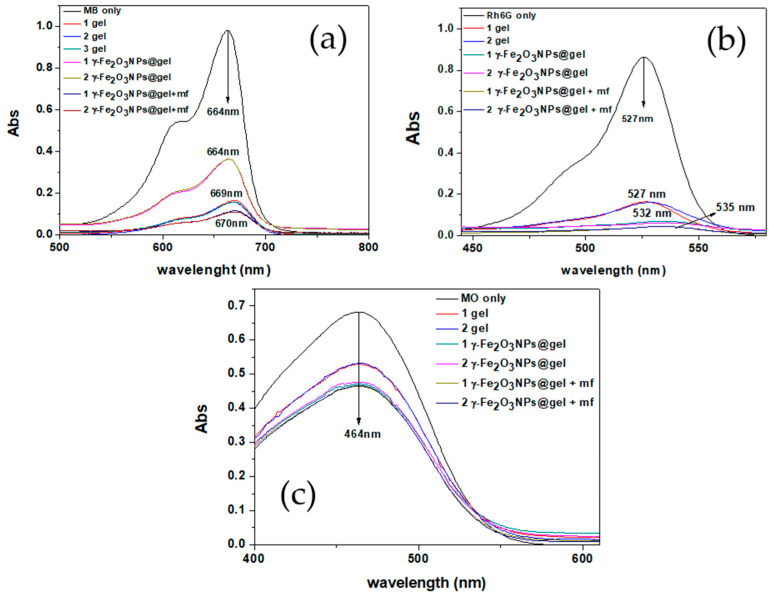
Absorbing abilities of gel, γ-Fe_2_O_3_NPs@gel, and γ-Fe_2_O_3_NPs@gel + mf for the removal of (**a**) MB (C_0_ = 5 ppm, pH = 7.4, 1 mg of adsorbent, flow rate = 0.2 mL/min); (**b**) Rh6G (C_0_ = 5 ppm, pH = 7.2, 1 mg of adsorbent, flow rate = 0.2 mL/min); (**c**) MO (C_0_ = 10 ppm, pH = 7.5, 1 mg of adsorbent, flow rate = 0.2 mL/min).

The absorbing abilities of native gel, γ-Fe_2_O_3_NPs@gel, and γ-Fe_2_O_3_NPs@gel + mf towards the selected dyes were tested with the experimental set-up employing syringes (Figure 5). Three different conditions were used, including (1) gravitational passage of the dye through the gels inside the syringe (Figure S2a); (2) gravitational passage with the application of filters attached to the outlet of the syringe (Figure S2b); and (3) the application of a constant pressure to increase the flow rate of the dye inside the syringe, combined with the use of a filter at the outlet of the syringe (Figure S2c). In terms of time and reproducibility, the third set-up was selected as the optimized condition for studying the absorbing abilities of the gels. It must be taken into account that the filters absorb part of the dye; therefore, this filter absorption was quantified for each dye and then subtracted from the amount of dye removed by each hydrogel system (see Figures S3–S6).

**Figure 6:**
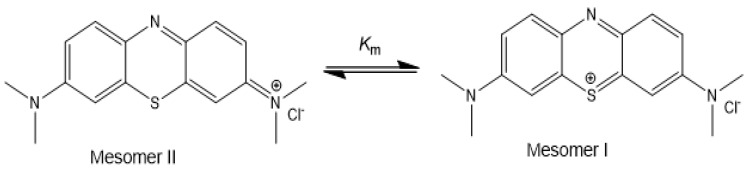
Alternative mesomeric structures for MB [41].

For the cationic dyes, MB and Rh6G, significant adsorptions (>60%) were observed for all adsorbents (gel, γ-Fe_2_O_3_NPs@gel and γ-Fe_2_O_3_NPs@gel + mf) (Table 3). For MB, the pristine gel showed a 20.3% higher RE% than the magnetogel; however, when the external magnet was used, the γ-Fe_2_O_3_NPs@gel + mf system demonstrated a 4.5% higher RE% than the native hydrogel. For both the γ-Fe_2_O_3_NPs@gel + mf and pristine gel systems, a red-shift was also detected, which may be attributed to the decrease in the dye’s concentration after passing through the gels. The UV-Vis spectrum of the initial MB solution shows a monomeric structure for the dye that can exist in two monomeric mesomers (I and II), based on data from the literature (see Figure 6) [42].

Based on Fernández-Pérez’s work on the mesomeric structure of MB in water, we propose that, after the removal of the dye by the native gel or γ-Fe_2_O_3_NPs@gel + mf, the purified solutions contain the mesomeric II structure of MB, which has different UV-Vis adsorption with the observed red-shift, compared to the initial MB solution.

For Rh6G, more than 95% of the dye was removed by both γ-Fe_2_O_3_NPs@gel and γ-Fe_2_O_3_NPs@gel + mf and, for the observed red-shift of the purified solutions, a monomer/dimer change in Rh6G may be proposed. In fact, the initial solution of Rh6G shows a combination of monomer/dimer species for the dye. After dilution, the concentration of monomers becomes significant and a spectral change is observed. Another important factor is the possible release of ions (e.g., Na^+^, Cl^−^) from the hydrogel matrix to the filtered solutions, which can also affect the spectral pattern on the dyes.

Conversely, regarding the cationic dyes, for MO, no significant spectral changes were detected after the filtration; the RE% values for MO are much lower (20–30%) compared to those for MB and Rh6G, probably due to the different charges in the dyes. Regarding the amount of MO adsorbed by the three hydrogel systems, γ-Fe_2_O_3_NPs@gel and γ-Fe_2_O_3_NPs@gel + mf show the same adsorption efficiencies (approximately 30%), compared to the lower amounts observed for the pristine gel (approximately 20%).

In Table 4, the adsorption capacities of γ-Fe2O3NPs@gel + mf (in the experimental set-up employing syringes) towards cationic dyes were compared with those of similar adsorbents described in the literature. The observed lower adsorption capacities for the magnetogels described in this study may be improved by modifying the formulation of the magnetogels, e.g., using cross-linkers that could also improve the mechanical stability of the magnetogels and, therefore, their reusability as adsorbents.

By comparing the two experimental setups (cuvettes and syringes, see Table 3), it can be seen that the adsorption using cuvettes is much lower, as it occurs only at the interface between the gel and solution, while in the case of syringes, the solution passes through the mass of the gel, allowing a greater interaction with it and, consequently, a greater adsorption of the dyes. More importantly, in each methodology, the trends of the RE% values of the adsorbents (Figures S7–S9) are changed.

## 3. Conclusions

Composite hydrogels have interesting adsorption properties and, in this study, we reported that the incorporation of magnetic NPs inside peptide-based hydrogels can be a valuable approach to additionally increase the adsorption capacities of hydrogels. In particular, the γ-Fe_2_O_3_NPs@gel + mf system was able to absorb up to 84% of MB and 97% of Rh6G from aqueous solutions containing 5 ppm of the dyes. However, for the anionic dye MO, the gels showed much lower adsorption capacities (lower than 30%) due to the dominant electrostatic repulsions between MO and the gel components.

The kinetic models of the cationic dyes mainly showed a physisorption mechanism for these adsorbents, while, for MO, both chemisorption and physisorption were observed, depending on the type of adsorbent. The results presented here support the application of peptide-based magnetogels in wastewater treatment. Future work should focus on scaling up materials’ preparation and testing them in practical scenarios.

## 4. Materials and Methods

### 4.1. Materials

L-Phenylalanyl-L-phenylalanine and *N*-(9-Fluorenylmethoxycarbonyl)-L-phenylalanine were obtained from Bachem GmbH (Weil am Rhein, Germany). All other chemicals, including FeCl_2_·4H_2_O, FeCl_3,_ and *Pseudomonas fluorescens* Lipase (≥20,000 U/mg) were purchased from Sigma-Aldrich (Saint Louis, MO, USA). All chemicals were employed without further purification. Ultra-pure water was prepared with a Zeneer Power I Scholar-UV (Full Tech Instruments, Rome, Italy) apparatus. The external magnetic field was generated by using a neodymium-based magnet (volume = 39.270 cm^3^, magnetization quality = N5, magnetic strength = 1.42–1.47 T).

### 4.2. Synthesis of γ-Fe_2_O_3_NPs@gel Magnetogels

Magnetogels were prepared with a two-step procedure, first preparing PAA-stabilized γ-Fe_2_O_3_NPs and then dispersing them in the aqueous solution of hydrogel precursors before gelation. The details of the synthetic procedures have been described previously [23].

### 4.3. SEM Analysis

SEM analyses were conducted with an Auriga field emission scanning electron microscope (Zeiss, Oberkochen, Germany), as previously described [23].

### 4.4. Rheological Measurements

The dynamo-mechanical analyses (mechanical spectroscopy) were studied for four samples including hydrogel and three magnetogels containing different concentrations of NPs. Rheological studies were carried out with an MCR 302 rotational rheometer (Anton Paar, Turin, Italy), as previously described [23].

### 4.5. Swelling Ability

Three mL of phosphate buffer solution (PBS, pH = 7.4) was placed on top of each hydrogel sample, followed by incubation at 30 °C in a thermostatic bath. After 24 h, the supernatants were removed and the samples were freeze-dried. The swelling degree was evaluated by using the following equation:

q = (W_s_ − W_d_)/W_d_ (1)

where q = swelling degree, W_s_ = weight of the gel after PBS removal, and W_d_ = weight of the freeze-dried gel.

### 4.6. Adsorption Experiments

For the cuvette experiments, gel and magnetogel samples were prepared in glass cuvettes. Then, 2 mL of aqueous dye solution (MB, Rh6G or MO) was cast on top of the gels. The concentration of MB and Rh6G was 5 ppm, while the MO concentration was 10 ppm.

UV-Vis spectroscopy was employed to monitor the absorbances of the solutions every 15 min. The removal efficiencies (RE) were calculated using Equation (2):

RE(%) = (C_0_ − C_f_)/C_0_ × 100 (2)

where C_0_ = initial dye concentration, C_f_ = dye concentration in the eluted solution.

Calibration curves were prepared for each dye and used for the calculations. The adsorption capacities (q_t_, mg g^−1^) of the adsorbents were calculated using Equation (3) [23]:

q_t_ = (C_0_ − C_t_)/m × V (3)

in which m (g) is the dried hydrogel mass, C_0_ and C_t_ (mg L^−1^) are the initial and equilibrium dye concentrations, and V (L) is the solution volume, respectively.

The kinetics of dye adsorption were analyzed with non-linear pseudo-first- and pseudo-second-order kinetic models (Equations (4) and (5)) [22].

log(q_e_ − q_t_) = logq_e_ − *k*_1_t/2.303 (4)

t/q_t_ = 1/*k*_2_q_e_^2^ + t/q_e_ (5)

## Figures and Tables

**Table 1:** Removal efficiencies and capacities (q_e_) of different hydrogel systems for MB, Rh6G, and MO.

	Adsorbent	Removal Efficiency% (RE%)	q_e_ (mg g^−1^)
MB	gel	30.8 ± 1	0.11 ± 0.0
	γ-Fe_2_O_3_NPs@gel	22.7 ± 2	0.08 ± 0.0
	γ-Fe_2_O_3_NPs@gel + mf	27.7 ± 1	0.09 ± 0.0
Rh6G	gel	45.7 ± 1	0.16 ± 0.0
	γ-Fe_2_O_3_NPs@gel	47.5 ± 1	0.17 ± 0.0
	γ-Fe_2_O_3_NPs@gel + mf	32.4 ± 2	0.11 ± 0.0
MO	gel	13.3 ± 1	0.09 ± 0.0
	γ-Fe_2_O_3_NPs@gel	14.1 ± 0	0.10 ± 0.0
	γ-Fe_2_O_3_NPs@gel + mf	13.4 ± 0	0.09 ± 0.0

**Table 2:** Kinetic data obtained from pseudo-first- and pseudo-second-order models for different hydrogel systems and MB, Rh6G, or MO at RT.

		Pseudo-First Order			Pseudo-Second Order		
	Adsorbent	k_1_ (min^−1^)	q_e_ (mg g^−1^)	R^2^	k_2_ (g mg^−1^ min^−1^)	q_e_ (mg g^−1^)	R^2^
MB	gel	0.011	0.14	0.9715	0.037	0.16	0.9713
	γ-Fe_2_O_3_NPs@gel	0.012	0.12	0.9493	0.0028	0.35	0.1329
	γ-Fe_2_O_3_NPs@gel + mf	0.011	0.15	0.9323	0.017	0.18	0.8257
Rh6G	gel	0.0074	0.17	0.992	0.023	0.23	0.9822
	γ-Fe_2_O_3_NPs@gel	0.0080	0.18	0.9881	0.026	0.23	0.9921
	γ-Fe_2_O_3_NPs@gel + mf	0.0089	0.14	0.9696	0.018	0.20	0.8597
MO	gel	0.0069	0.10	0.9712	0.048	0.13	0.954
	γ-Fe_2_O_3_NPs@gel	0.010	0.13	0.9328	0.037	0.15	0.9832
	γ-Fe_2_O_3_NPs@gel + mf	0.0080	0.11	0.9638	0.024	0.15	0.8149

**Table 3:** Adsorption efficiencies of different hydrogel systems for the removal of MB, Rh6G, or MO from an aqueous phase using the experimental set-up employing syringes.

	Adsorbent	RE% of Cuvette Set-Up	RE% of Syringe Set-Up
MB	gel	30.8 ± 1	80.4 ± 1
	γ-Fe_2_O_3_NPs@gel	22.7 ± 2	60.1 ± 2
	γ-Fe_2_O_3_NPs@gel + mf	27.7 ± 1	84.9 ± 0
R6G	gel	45.7 ± 1	83.2 ± 1
	γ-Fe_2_O_3_NPs@gel	47.5 ± 1	95.0 ± 0
	γ-Fe_2_O_3_NPs@gel + mf	32.4 ± 2	97.6 ± 0
MO	gel	13.3 ± 1	21.8 ± 7
	γ-Fe_2_O_3_NPs@gel	14.1 ± 0	30.0 ± 6
	γ-Fe_2_O_3_NPs@gel + mf	13.4 ± 0	31.3 ± 6

**Table 4:** Comparison of the adsorption capacities of γ-Fe2O3NPs@gel + mf with similar adsorbents for the removal of cationic dyes.

Adsorbent	Cationic Dyes	C_0_ (mg mL^−1^)	Adsorption Capacities of Syringe Set-Up (mg g^−1^)	Reference
γ-Fe_2_O_3_NPs@gel + mf	MB	0.005	0.9	this work
γ-Fe_2_O_3_NPs@gel + mf	Rh6G	0.005	1.1	this work
poly(acrylic acid-acrylamide-butyl methacrylate) magnetic hydrogel	MB	50–100	12.6	[43]
Fe_3_O_4_/poly(2-hydroxyethyl methacrylate-co-itaconic acid) magnetic hydrogels	MB	0.05–0.2	174.9	[44]
poly(2-(2-methoxyethoxy) ethyl methacrylate-*co*-oligo (ethylene glycol) methacrylate-*co*-acrylic acid) (PMOA) hydrogel-magnetic attapulgite/Fe_3_O_4_	RhB (rhodamine B)	0.001	1.65	[45]

## Data Availability

The data presented in this study are available in the article.

## Supplementary Materials

The following supporting information can be downloaded at: https://www.mdpi.com/article/10.3390/gels10050287/s1, Materials and Methods; Figure S1: (a) Calibration curve of methylene blue (MB); (b) calibration curve of rhodamine 6G (R6G); (c) calibration curve of methyl orange (MO).; Figure S2: (a) Gravitational passage of MB through the gels inside the syringe; (b) gravitational passages with applying the filter in the outlet of the syringe; (c) gravitational passage with applying the filter and a constant pressure to increase the flow rate of the dye inside the syringe.; Figure S3: (a) Absorption of MB by the filter for different cycles; (b) amount of MB adsorbed by the filter in each cycle; (c) visual observation of the MB adsorption by the filter.; Figure S4: (a) Absorption of Rh6G by the filter for different cycles; (b) amount of Rh6G adsorbed by the filter in each cycle; (c) visual observation of the Rh6G adsorption by the filter.; Figure S5: (a) Absorption of MO by the filter for different cycles; (b) amount of MO adsorbed by the filter in each cycle; (c) visual observation of the MO adsorption by the filter. Figure S6: (a) Separation of the dyes by gel, γ-Fe_2_O_3_NPs@gel, and (γ-Fe_2_O_3_NPs@gel + mf) using the syringe with filter and external pressure for (a) MB; (b) Rh6G; (c) and MO.; Figure S7: Comparison of the cuvette and syringe methodology for the MB adsorption by (a) gel, (b) γ-Fe_2_O_3_NPs@gel, and (c) γ-Fe_2_O_3_NPs@gel + mf.; Figure S8: Comparison of the cuvette and syringe methodology for the Rh6G adsorption by (a) gel, (b) γ-Fe_2_O_3_NPs@gel, and (c) γ-Fe_2_O_3_NPs@gel + mf.; Figure S9: Comparison of the cuvette and syringe methodology for the MO adsorption MO by (a) gel, (b) γ-Fe_2_O_3_NPs@gel, and (c) γ-Fe_2_O_3_NPs@gel + mf.
