# Supplementary figures and images for: Preparation of Peptide-Based Magnetogels for Removing Organic Dyes from Water
Source: Gels. 2024 Apr 24;10(5):287. doi: 10.3390/gels10050287 (PMC11120949; doi:10.3390/gels10050287)

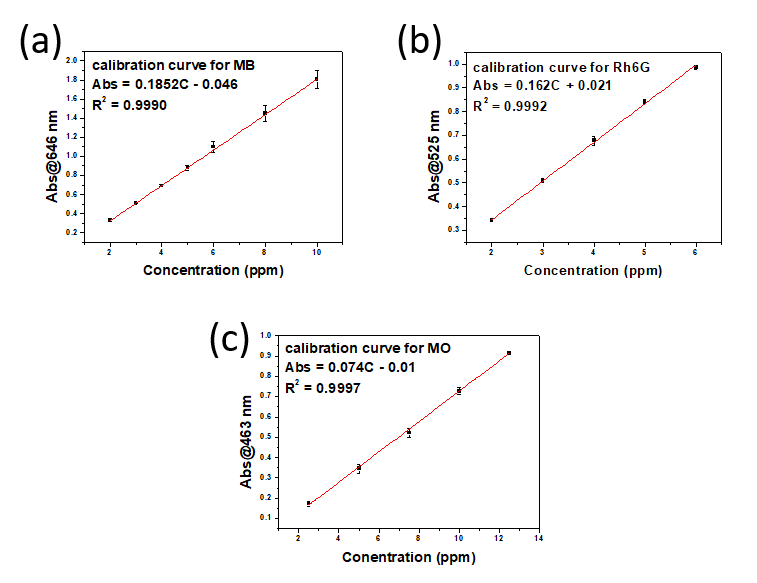

Supplement: Supplementary file 1 [file gels-10-00287-s001.zip › Figure S1.png]

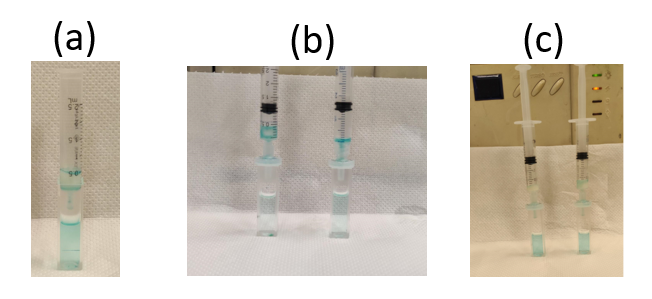

Supplement: Supplementary file 1 [file gels-10-00287-s001.zip › Figure S2.png]

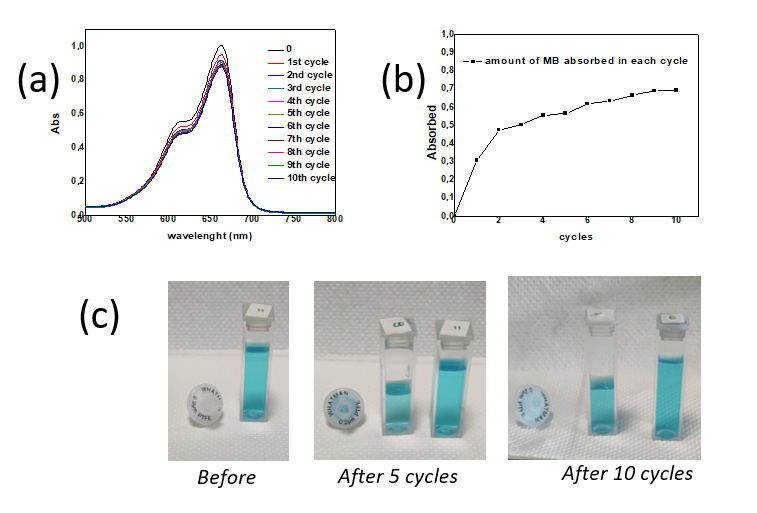

Supplement: Supplementary file 1 [file gels-10-00287-s001.zip › Figure S3.png]

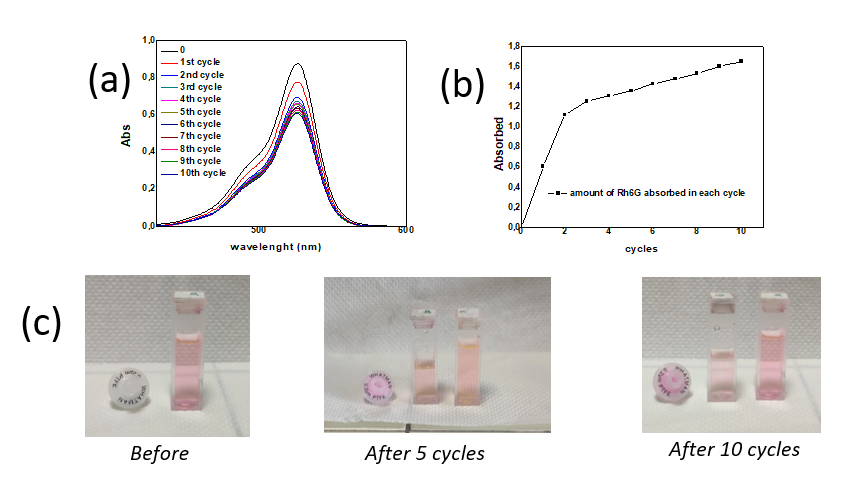

Supplement: Supplementary file 1 [file gels-10-00287-s001.zip › Figure S4.png]

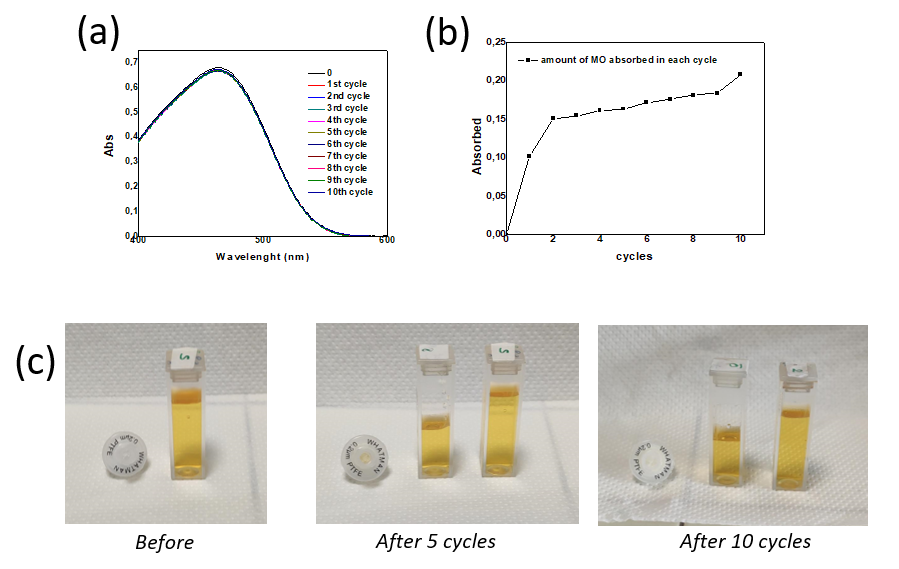

Supplement: Supplementary file 1 [file gels-10-00287-s001.zip › Figure S5.png]

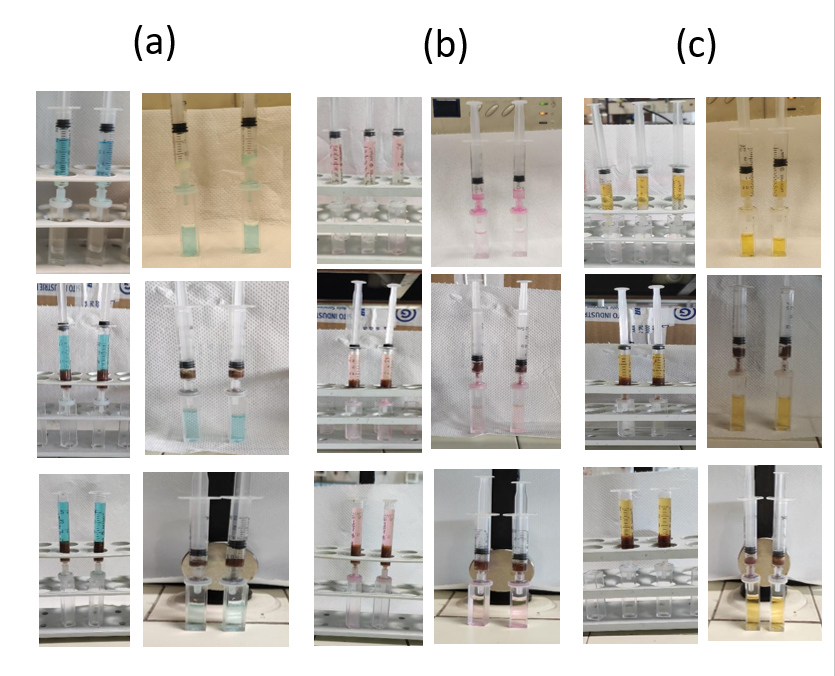

Supplement: Supplementary file 1 [file gels-10-00287-s001.zip › Figure S6.png]

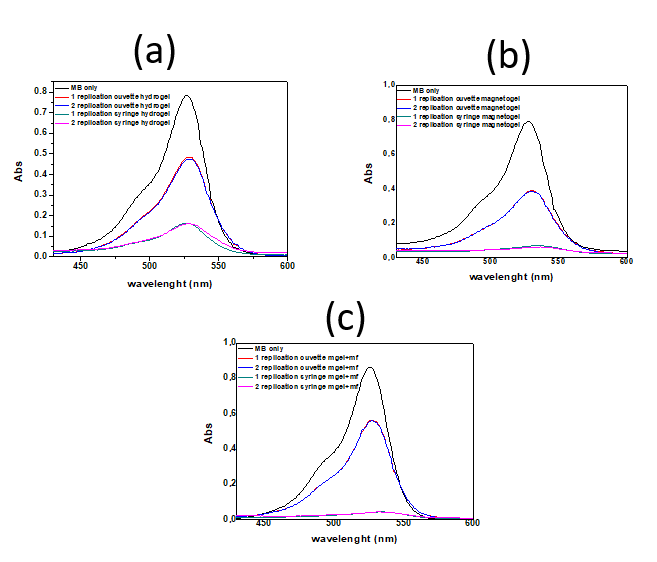

Supplement: Supplementary file 1 [file gels-10-00287-s001.zip › Figure S7.png]

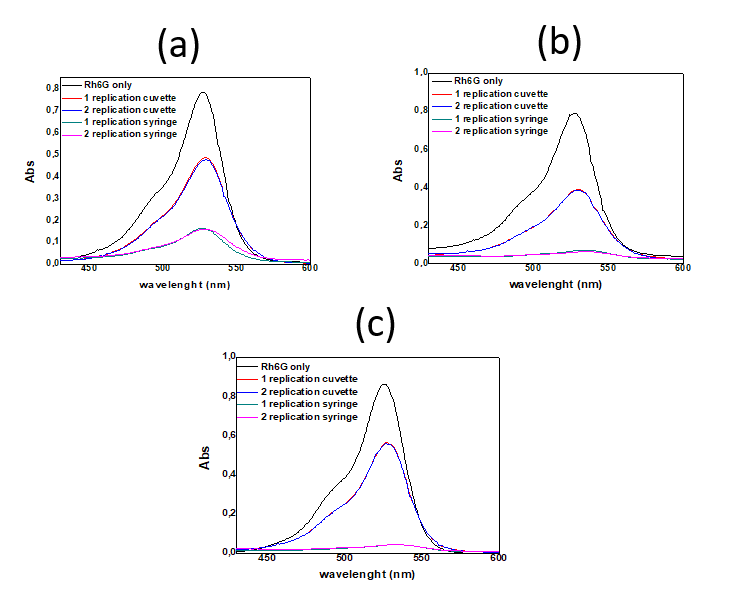

Supplement: Supplementary file 1 [file gels-10-00287-s001.zip › Figure S8.png]

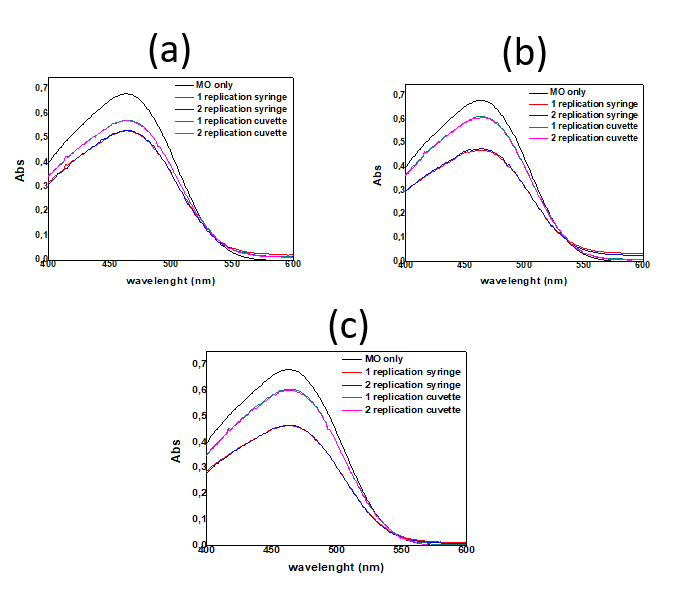

Supplement: Supplementary file 1 [file gels-10-00287-s001.zip › Figure S9.png]
